# Trends in breastfeeding and complementary feeding practices in Pakistan, 1990-2007

**DOI:** 10.1186/1746-4358-6-15

**Published:** 2011-10-21

**Authors:** Hafsa Muhammad Hanif

**Affiliations:** 1 Dow Medical College, Dow University of Health Sciences, Baba-e-Urdu Road, Karachi 74400, Pakistan

**Keywords:** breastfeeding, complementary feeding, infant and young child feeding indicators, Pakistan

## Abstract

**Background:**

Breastfeeding and complementary feeding practices have profound implications for the maternal and child health status of a society. Feeding practices in Pakistan are suboptimal, leading to adverse outcomes on child health. In Pakistan, the Maternal, Neonatal and Child Health (MNCH) Program, in collaboration with several international organizations, including WHO and UNICEF, is working to improve these feeding practices in the country. The aim of this paper is to evaluate the effectiveness of these programs.

**Methods:**

Estimates on the various indicators for infant and young child feeding proposed by WHO were analyzed in light of the Pakistan Demographic and Health Surveys (1990-91 and 2006-07) and several other national studies conducted since 1995.

**Results:**

Nearly half the core and optional indicators have improved over the years, though modestly; the others have demonstrated no statistically significant improvement over the years. Of the five indicators required in the WHO tool for the assessment of infant and young child feeding, introduction of complementary foods, bottle-feeding, and early initiation of breastfeeding, stand in the poor category, while exclusive breastfeeding and duration of breastfeeding fall in the fair category, suggesting an overall poor status.

**Conclusions:**

There is considerable scope to improve breastfeeding and complementary feeding in Pakistan. Further programs should focus on improving the following indicators that have shown no significant development: early initiation of breastfeeding, exclusive breastfeeding under six months, continued breastfeeding at two years, age appropriate feeding, and bottle feeding. Effective implementation of interventions that are known to improve breastfeeding practices is imperative, as is further research to yield data that can lead future endeavors.

## Background

Breastfeeding and complementary feeding practices have long been demonstrated to have significant implications for maternal and child health. Healthy breastfeeding practices reduce child mortality and morbidity, and improve immunity in children, besides being essential for their optimal growth and development [1]. In mothers, breastfeeding is associated with enhanced emotional attachment to the infant, reduced risk of breast and endometrial cancer, increased duration of post partum amenorrhea, and consequent birth spacing, as well as several other health benefits [1]. The World Health Organization recommends that infants be exclusively breastfed for the first six months, followed by breastfeeding along with complementary foods for up to two years of age or beyond [2].

Even though nearly all children in Pakistan are breastfed [3], feeding practices are suboptimal, leading to adverse outcomes on child health, worsening the already poor state of child health and nutrition in the country, and overburdening the meager health coverage. Hence, improving breastfeeding and infant feeding practices is an important means used by the World Health Organization to improve child health in Pakistan and other developing countries [4]. In fact, exclusive breastfeeding for the first six months of postnatal life has been identified as the single most instrumental intervention that can aid in decreasing child mortality and morbidity [5].

In Pakistan, the Maternal, Neonatal and Child Health (MNCH) Program, Ministry of Health, in collaboration with WHO, UNICEF and USAID, is working towards the improvement of the health status indicators of the country. Breastfeeding and complementary feeding practices are among the many important issues that the Program addresses [6] through training and the network of community workers and health centers.

The objective of this article is to present the trends of breastfeeding and complementary feeding practices in Pakistan since the 1990s in order to assess the degree of effectiveness of the efforts of the MNCH Program, WHO, various NGOs, and other stakeholders in improving feeding practices, and the extent to which they have achieved their objectives. Despite the concern that the issue has received over the years, data on the subject from a single source is lacking. Even in surveys that do have relevant data, several indicators have not been precisely described, and require additional analysis of underlying data to calculate. Hence estimates of feeding practices in collected data, and in comparison to other sources, is timely.

## Methods

The estimates presented in the following research publications were studied: Pakistan Demographic and Health Survey, 1990-1991 [7] [DHS 90-91]; Pakistan Demographic and Health Survey, 2006-2007 [3] [DHS 06-07]; Pakistan Integrated Household Survey, 1998-1999 [8] (Federal Bureau of Statistics) [PIHS 98-99]; Pakistan Integrated Household Survey, 2001-2002 [9] (Federal Bureau of Statistics) [PIHS 01-02]; Pakistan Social and Living Standards Measurement Survey, 2005-2006 [10] (Federal Bureau of Statistics) [PSLM 05-06]; and Pakistan Social and Living Standards Measurement Survey, 2007-2008 [11] (Federal Bureau of Statistics) [PSLM 07-08]. See Table 1.

**Table 1:** Summary of surveys

Title of survey	Conducted by	Coverage	Sample size	Sampling method
Pakistan Demographic and Health Survey, 1990-91 [7]	National Institute of Population Studies (Pakistan), and Macro International Inc.	National	7193 households	Unspecified
Pakistan Integrated Household Survey, 1998-99 [8]	Federal Bureau of Statistics, Pakistan	National	16305 households	Multi-stage stratified random sample
Pakistan Integrated Household Survey, 2001-02 [9]	Federal Bureau of Statistics, Pakistan	National	16182 households	Multi-stage stratified random sample
Pakistan Social and Living Standards Measurement Survey, 2005-06 [10]	Federal Bureau of Statistics, Pakistan	National	15453 households	Multi-stage stratified random sample
Pakistan Demographic and Health Survey, 2006-07 [3]	National Institute of Population Studies (Pakistan) and Macro International Inc.	National	97, 687 households	Multi-stage stratified random sample
Pakistan Social and Living Standards Measurement Survey, 2007-08 [11]	Federal Bureau of Statistics, Pakistan	National	15512 households	Multi-stage stratified random sample

The indicators for breastfeeding and complementary feeding practices have been adapted from infant and young child feeding indicators recommended by the World Health Organization [12], and the trends presented below are based on the aforementioned publications. Where data were available, 95% confidence intervals were calculated for the percentages using the standard formula.

The purpose of this article is not to emulate the detailed definitions and implications of each of the indicators, nor to present their underlying rationales. Readers can access this information in the World Health Organization publication, 'Indicators for assessing infant and young child feeding practices' (Parts I and III) [13,14].

## Results

Table 2 presents the infant and young child feeding indicators as proposed by the World Health Organization [12]. The indicators are classified as 'core' and 'optional', with the following being the most important among the core indicators: early initiation of breastfeeding; exclusive breastfeeding under six months; minimum acceptable diet; and consumption of iron-rich or iron-fortified foods. Estimates on the trends of the latter two of these four, along with two other core indicators, namely, minimum dietary diversity and meal frequency, were not given in any publication studied, nor could they be found in other national surveys. Trends of the remaining indicators are presented below.

**Table 2:** Indicators for Infant and Young Child Feeding (WHO) [reproduced with permission from EUPHIX: European Union Public Health Information and Knowledge System]

Core indicators	Optional indicators
**1. Early initiation of breastfeeding:**	**9. Children ever breastfed:**
Proportion of children born in the last 23.9 months who were put to the breast within one hour of birth.	Proportion of children born in the last 23.9 months who were ever breastfed.
**2. Exclusive breastfeeding under six months:**	**10. Continued breastfeeding at two years:**
Proportion of infants 0-5.9 months of age who are fed exclusively with breast milk.	Proportion of children 20-23.9 months of age who are fed breast milk.
**3. Continued breastfeeding at one year:**	**11. Age-appropriate breastfeeding:**
Proportion of children 12-15.9 months of age who are fed breast milk.	Proportion of children 0-23.9 months of age who are appropriately breastfed.
**4. Introduction of solid, semi-solid or soft foods:**	**12. Predominant breastfeeding under six months:**
Proportion of infants 6-8.9 months of age who receive solid, semi-solid or soft foods.	Proportion of infants 0-5.9 months of age who are predominantly breastfed.
**5. Minimum dietary diversity:**	**13. Duration of breastfeeding:**
Proportion of children 6-23.9 months of age who receive foods from four or more out of seven food groups.	Median duration of breastfeeding among children 0-35.9 months of age.
**6. Minimum meal frequency:**	**14. Bottle feeding:**
Proportion of breastfed and non-breastfed children 6-23.9 months of age who receive solid, semi-solid or soft foods (including milk feeds for non-breastfed children) the minimum number of times or more.	Proportion of children 0-23.9 months of age who are fed with a bottle.
**7. Minimum acceptable diet:**	**15. Milk feeding frequency for non-breastfed children:**
Proportion of children 6-23.9 months of age who receive a minimum acceptable diet (apart from breast milk).	Proportion of non-breastfed children 6-23.9 months of age who receive at least two milk feedings (infant formula, cow's milk or other animal milk).
**8. Consumption of iron-rich or iron-fortified foods:**	
Proportion of children 6-23.9 months of age who receive an iron-rich or iron-fortified food that is specially designed for infants and young children, or that is fortified in the home.	

**Note**:

Indicators 2-8, 10-12 and 14-15 are based on a 24-hour recall period.

Indicators 1, 2, 7 and 8 are considered top priorities for reporting among the core indicators.

Indicator 2 can be disaggregated for ages 0-1, 2-3, 4-5 and 0-3 months.

The seven food groups mentioned under indicator 5 are: grains, roots and tubers; legumes and nuts; dairy products (milk, yogurt, cheese); flesh foods (meat, fish, poultry and liver/organ meats); eggs; vitamin A rich fruits and vegetables; other fruits and vegetables.

Minimum number of times mentioned under indicator 6 is defined as: two times for breastfed infants 6-8.9 months; three times for breastfed children 9-23.9 months; four times for non-breastfed children 6-23.9 months.

Indicator 7 is the sum of two fractions: (1) the proportion of breastfed children 6-23.9 months of age who had at least the minimum dietary diversity and the minimum meal frequency during the previous day; plus (2) the proportion of non-breastfed children 6-23.9 months of age who received at least two milk feedings and had at least the minimum dietary diversity and the minimum meal frequency during the previous day.

Indicator 11 is the sum of exclusive breastfeeding under six months plus the proportion of children 6-23.9 months of age who received breast milk as well as solid, semi-solid or soft foods during the previous day.

The calculations for estimates that were not directly reported in the publications, but had to be calculated using the available data, are presented as an additional file [see Additional file 1].

### I. Early initiation of breastfeeding

The World Health Organization recommends that breastfeeding be started soon after birth, preferably within the first hour [2]. This improves breastfeeding success [15], the health and survival status of newborns, and the emotional attachment of the child to the mother [16]. Early initiation of breastfeeding is the also among the four top priority core indicators. In Pakistan, although breastfeeding is nearly universal, early initiation rates are not as high. In 1990-91, among the last born children, those for whom breastfeeding was initiated within the first hour and day of delivery stood at 8.5% (95% CI: 7.6, 9.4) and 25.8% (95% CI: 24.5, 27.1) respectively [7]. The corresponding estimates in 2006-07 demonstrate marked improvement, with rates of 27.2% (95% CI: 26.0, 28.3) and 65.5% (95% CI: 64.3, 66.8) respectively [3].

### II. Exclusive breastfeeding under six months

The proportion of infants under six months who were being exclusively breastfed was 22.8% (95% CI: 19.8-25.7) in 1990-91 [7], which had increased to 37.1% (95% CI: 34.0, 40.2) by 2006-07 [3].

### III. Continued breastfeeding at one year

The percentage of infants over 12 months and under 16 months who were continuing to breastfeed increased slightly from 78.2% (95% CI: 74.7, 81.7) in 1990-91 [7] to 79% (95% CI: 75.8, 82.2) in 2006-07 [3]. The change, however, is not statistically significant.

### IV. Introduction of solid, semi-solid or soft foods

**Figure 1:**
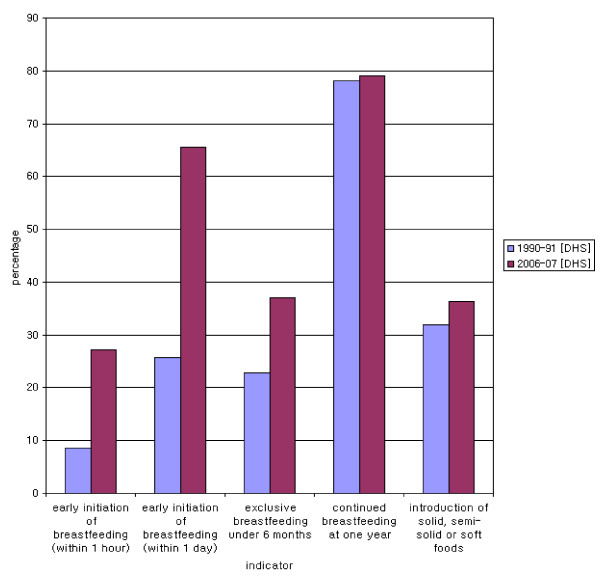
**Core indicators from Pakistan Demographic and Health Surveys 1990-91 and 2006-07**.

The proportion of infants aged 6 to 9 months who received solid/semi solid or soft food as a supplement was 32.1% (95% CI: 27.5, 36.7) [7] and 36.3% (95% CI: 32.4, 40.2) [3] in 1990-91 and 2006-07 respectively, a statistically insignificant difference. (See Figure 1).

### V. Children ever breastfed

Of all children born in the five years preceding the survey, the proportion who were ever breastfed was 93.5% (95% CI: 92.9, 94.1) in 1990-91 [7], which was similar to the corresponding value in 2006-07, 94.3% (95% CI: 93.8, 94.8) [3]. The percentage of women who breastfed their last child fell from 96% in 1998-99 [PIHS] to 92% in 2001-02 [PIHS 01-02]. According to the estimates of PSLM, the percentage was 96% in 2005-06, and remained stable till 2007-08. (See Table 3).

**Table 3:** Children ever breastfed

Survey	Percentage	95% Confidence Interval
DHS 1990-91	93.5	92.9, 94.1
DHS 2006-07	94.3	93.8, 94.8
PIHS 1998-99	96	N/A*
PIHS 2001-02	92	N/A
PSLM 2005-06	96	N/A
PSLM 2007-08	96	N/A

N/A: Not available (unable to calculate because data are not available)

### VI. Continued breastfeeding at two years

Continued breastfeeding, in terms of the percentage of children 20-23 months of age breastfeeding, also did not change: 51.7% (95% CI: 45.9, 57.4) in 1990-91 [7] and 54.9% (95% CI: 49.9, 59.9) in 2006-07 [3].

### VII. Age appropriate breastfeeding

Age appropriate breastfeeding is defined as the sum of the proportion of infants less than 6 months who are exclusively breastfed, and those between 6-24 months who are receiving a complementary diet along with breast milk. The prevalence of age appropriate breastfeeding did not change in the approximately 15-year period between the two DHS surveys: 48.2% (95% CI: 46.2, 50.1) in 1990-91 and 46.2% (95% CI: 44.5, 47.9) in 2006-07.

### VIII. Predominant breastfeeding under 6 months

Predominant breastfeeding occurs when breast milk is the predominant source of nourishment for the infant, but the infant additionally receives water, water based drinks, and fruit juice [17]. The proportion of infants under 6 months of age who were predominantly breastfed in 2006-07 was 18.8% (95% CI: 16.3, 21.2) [3]. Sufficient data to calculate the corresponding value in the 1990-91 survey are not available. However, the proportion of infants under 6 months who received *only *plain water in addition to breast milk, which to some degree is an indicator of the predominant feeding status, was 11.0% (95% CI: 8.7, 13.2) in 1990-91 [7], which increased to 17.1% (95% CI: 14.8, 19.5) by 2006-07 [3].

### IX. Duration of breastfeeding

The mean duration of *any *breastfeeding (of all the children born in the five years preceding the survey) decreased from 19.8 months in 1990-91 to 18.3 in 2006-07 (of all the children born in the three years preceding the survey), while the duration of exclusive breastfeeding increased from 2.9 to 3.2, and that of predominant/full breastfeeding from 4.9 to 5.6 months.

### X. Bottle feeding

**Figure 2:**
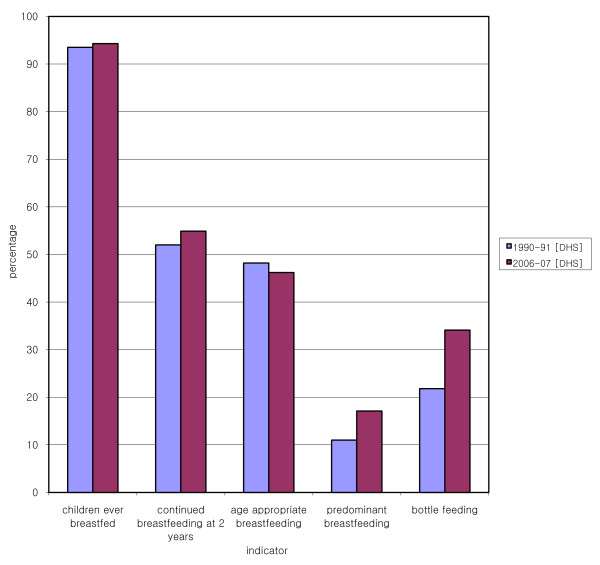
**Optional indicators from Pakistan Demographic and Health Surveys 1990-91 and 2006-07**.

The use of pacifiers and bottles with nipples has been shown to interfere with successful breastfeeding [18], leading to reduced duration of breastfeeding, [19] and 'nipple confusion' in infants [20]. In third world countries such as Pakistan, bottle-feeding poses the additional risk of introducing pathogens into the infant, because of unhygienic practices during handling and preparation leading to increased susceptibility to diarrhea and infections [21,22]. The Baby Friendly Hospital Initiative of the World Health Organization specifically discourages giving artificial 'teats or pacifiers' to breastfeeding infants in its 'Ten steps to successful breastfeeding' [23]. The percentage of children under 24 months of age who were fed using a bottle with a nipple in 2006-07 was 34.1% (95% CI: 20.0, 23.5) [3]. Corresponding estimates are not given in the earlier survey. However, the prevalence of bottle feeding among breastfeeding children under 24 months, which may to some degree be an indicator of the prevalence of bottle feeding in all children (breastfeeding and not breastfeeding), was 21.8% (95% CI: 32.5, 35.7). (See Figure 2).

### XI. Milk feeding frequency for non-breastfed children

The World Health Organization requires that non-breastfeeding infants aged 6-24 months be given at least two milk feeds daily [24]. Milk feeding frequency however was not reported in any publication. But the percentage of non-breastfed children in the specified age group who were fed infant formula/other milk in the day or night preceding the interview was 91.9% in 2006-07 [3], while the data in the earlier DHS survey were insufficient to calculate this.

## Discussion

The results presented here show trends in the core and optional indicators. While early initiation and exclusive breastfeeding have improved, continued breastfeeding at the first year, and the introduction of solid, semi-solid and soft foods have experienced no statistically significant change.

Data on five core indicators, namely: minimum acceptable diet, consumption of iron-rich or iron-fortified foods, minimum dietary diversity, and meal frequency, is lacking in the surveys studied, nor could it be retrieved from any other publication. The National Institute of Population Studies should consider including this information in future Demographic and Health Surveys so a more accurate analysis of the indicators of breastfeeding and complementary feeding can be made possible. The Federal Bureau of Statistics may also like to consider this in its future relevant surveys.

The WHO optional indicators (continued breastfeeding at two years, age appropriate breastfeeding and the duration of breastfeeding) demonstrate decreased prevalence, while bottle feeding has increased. This suggests that these aspects of healthy breastfeeding require more concern. In order to achieve optimal results, it seems imperative for the MNCH program, WHO, UNICEF and other organizations working for the promotion of healthy breastfeeding practices to consider placing more emphasis on these aspects in their future workshops, training and other endeavors.

It is noteworthy that the confidence intervals for the indicators were calculated assuming a simple random mode of sampling. In DHS however, a multi-stage stratified sample was used, so the actual error margin is likely to be higher because of the principle of 'design effect' [3]. Hence, the apparent improvement in some indicators is likely to be even more modest.

**Figure 3:**
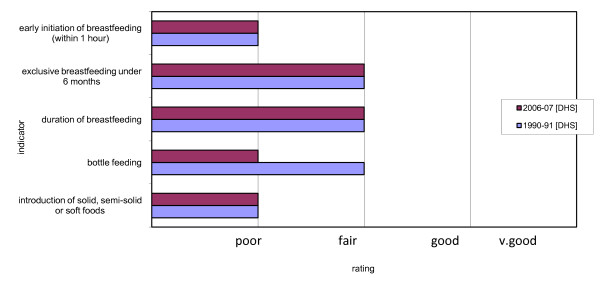
**Indicator ratings from Pakistan Demographic and Health Surveys 1990-91 and 2006-07**.

The five indicators required in the WHO tool for the assessment of national practices policies and programs related to infant and young child feeding [4], displayed in Figure 3, show no improvement over time. Furthermore, the rating for bottle feeding has gone down from 'fair' to 'poor'. Also noteworthy is that three of the five indicators fall in the poor category, which suggests there is considerable scope for improving breastfeeding and complementary feeding practices. In one independent assessment of IYCF policies, practices and programs, Pakistan ranks 4^th ^among eight other countries in the region, including India, Afghanistan, Sri Lanka, Nepal, Bangladesh, Bhutan and Maldives, with a score of 75.5 out of 150 (Grade C), signifying low achievement [25].

In the backdrop of the economic crises that has loomed over the country, the recent devastating floods, and the ensuing striking rates of malnutrition, promotion of healthy breastfeeding practices has assumed an even more pivotal role. An enhanced cooperation among national and international NGOs and the government is crucial to addressing the issue. Interventions that are known to improve breastfeeding practices are not being effectively exploited; influencing public and private sector hospital policies, in particular, needs to be aggressively addressed. Furthermore, despite its import, research on the subject is lacking, and should be promoted to generate reliable data that can be used to guide the effective planning and development of interventions.

## Conclusions

Data on breastfeeding indicators in Pakistan suggest an overall unsatisfactory status. Hence, there is much scope for improvement. Further programs should focus on improving the following indicators that have shown no significant development: early initiation of breastfeeding, exclusive breastfeeding under six months, continued breastfeeding at two years, age appropriate feeding, and bottle feeding. Effective implementation of interventions that are known to improve breastfeeding practices is imperative, as is further research to yield data that can lead future endeavors.

## List of abbreviations

PDHS: Pakistan Demographic and Health Survey; PIHS: Pakistan Integrated Household Survey (Federal Bureau of Statistics); PSLM: Pakistan Social and Living Standards Measurement Survey (Federal Bureau of Statistics)

## Competing interests

The author declares that they have no competing interests.

## Supplementary Material

Additional file 1 **Calculations**. Calculations to estimate indicators of breastfeeding and complementary feeding practices. Click here for file
