# Supplementary material for: Trends in breastfeeding and complementary feeding practices in Pakistan, 1990-2007
Source: Int Breastfeed J. 2011 Oct 21;6:15. doi: 10.1186/1746-4358-6-15 (PMC3207870; doi:10.1186/1746-4358-6-15)
Supplement: Additional file 1 — Calculations. Calculations to estimate indicators of breastfeeding and complementary feeding practices. [file 1746-4358-6-15-S1.DOCX]

**CALCULATIONS**

- Percentage of children ever breastfed = 94.3 [2]

Number of last-born children ever breastfed = 5,369

Total no. of last born children (approx.) = (100 ÷ 94.3) x 5,369 = 5693.53

Number of last born children who were initiated breastfeeding within the first hour of birth = (28.8 ÷100) x 5369 = 1546.272

**%age of last born children who were initiated breastfeeding within the first hour of birth**

**=** (1546.272 ÷ 5693.53) x 100

**=27.2%**

Number of last born children who were initiated breastfeeding within the first day of birth = (69.5 ÷ 100) x 5369 = 3731.455

**%age of last born children who were initiated breastfeeding within the first day of birth**

**=** (3731.455 ÷ 5693.53) x 100

**= 65.5%**

- total no. of infants under 6 months = 237+272+253 = 762 [1]

**%age of infants under 6 months who were exclusively breast fed** =

{[(no. of exclusively breastfed infants of 0-1 months) + (no. of exclusively breastfed infants of 2-3 months) + (no. of exclusively breastfed infants of 4-5 months)] ÷ 762} x100

= {[(0.272x237) + (0.237x272)+ (0.176x253)] ÷ 762} x 100 [1]

= **22.8%**

- total no. of infants over 12 and under 16 months = 279+265 = 544 [1]

**%age of infants over 12 and under 16 months who were still breastfeeding** =

100 - %age of infants over 12 and under 16 years who were NOT breastfeeding =

100 – [(14x279 + 30x265) ÷ 544] [1]

= **78.2%**

- total no. of infants over 6 months and under 9 months = 208 + 191 = 399 [1]

no. of infants over 6 months and under 9 months receiving solid, semi-solid or soft foods = 0.299x208 + 0.345x191 = 128.087

**%age of infants over 6 months and under 9 months receiving solid, semi-solid or soft foods =**

(128.087÷399) x 100

= **32.1%**

- total no. of children over 20 months and under 24 months = 142 + 148 = 290 [1]

no. of infants under 24 months still breastfeeding = 0.562x142 + 0.473x148 = 149.808 [1]

**%age of infants over 20 months and under 24 months still breastfeeding** =

(149.808 ÷ 290) x 100

= 51.7%

- total no. of children under 24 months=

237+272+253+235+209+164+279+265+184+197+142+148 = 2585 [1]

no. of children under 6 months being exclusively breastfed = 0.272x237 + 0.237x272 + 0.176x253 = 173.456 [1]

no. of children 6-24 months old being appropriately breastfed = 0.594x235 + 0.590x209 + 0.650x164 + 0.719 x279 + 0.592x265 + 0.575x184 + 0.537x197 + 0.508x142 + 0.410x148 = 1071.386 [1]

**%age of children under 24 months appropriately breastfed** = [(173.456+1071.386) ÷ 2585] x 100

= **48.2%**

total no. of children under 24 months = 284+359+319+447+373+916+606 = 3304 [2]

no. of children under 6 months being exclusively breastfed = 0.546x284 + 0.357x359 + 0.231x319 = 356.916 [2]

no. of children 6-24 months old being appropriately breastfed = 0.336x447 + 0.517x373 + 0.581x916 + 0.486 x606 = 1169.745 [2]

**%age of children under 24 months appropriately breastfed** = [(356.916+1169.745) ÷ 3304] x 100

= **46.2%**

- total no. of infants under 6 months = 284+359+319 = 962 [2]

no. of predominantly breastfed infants under 6 months = (0.128+0.011)x284 + (0.184+0.014)x359 + (0.196+0.023)x319 = 180.419 [2]

**%age of predominantly breastfed children under 6 months** = (180.419 ÷ 962) x 100

= **18.8%**

- total no. of infants under 6 months = 237+272+253 = 762 [1]

no. of infants under 6 months breastfeeding and receiving plain water only = 0.115x237 + 0.097x272 + 0.118x253 = 83.493 [1]

**%age of infants under 6 months breastfeeding and receiving plain water only** = (83.493 ÷ 762) x 100

= **11.0%**

- total no. of infants under 6 months = 284+359+319 = 962 [2]

no. of infants under 6 months breastfeeding and receiving plain water only = 0.128x284 + 0.184x359 + 0.196x319 = 164.932 [2]

**%age of infants under 6 months breastfeeding and receiving plain water only** = (164.932 ÷ 962) x 100

= **17.1%**

- total no. of children under 24 months = 284+359+319+447+373+916+606 = 3304 [2]

no. of children using a bottle with a nipple = 0.168x284 + 0.297x359 + 0.332x319 + 0.345x447 + 0.402x373 + 0.378x916 + 0.357x606 = 1126.994 [2]

**percentage of children under 24 months using a bottle with a nipple** =

(1126.994 ÷ 3304) x 100

= **34.1%**

- total no. of breastfeeding children under 24 months =

228+263+236+208+191+134+240+185+111+117+80+70 = 2063 [1]

no. of breastfeeding children using a bottle with a nipple = 16.9x228 + 30.8x263 + 29.1x236 + 25.7x208 + 26.2x191 + 23.9x134 + 19.3x240 +18.6x185 + 5.3x111 + 19.3x117 + 14.3x80 + 6.7x70 = 44906 [1]

**percentage of children under 24 months using a bottle with a nipple** =

(44906 ÷ 2063) x 100

= **21.8%**

- total no. of non-breastfeeding children aged 6-24 months =

44+57+207+204= 512 [2]

no. of non-breastfeeding children aged 6-24 months who were fed infant formula/other milk in the 24 hour period preceding the survey = 0.985x44 + 0.884x57 + 0.967x207 + 0.866x204 = 470.561 [2]

**%age of non-breastfeeding children aged 6-24 months who were fed infant formula/other milk in the 24 hour period preceding the survey** =

(470.561 ÷ 512) x 100

= **91.9%**

**REFERENCES**

1. National Institute of Population Studies (NIPS) [Pakistan], and Macro International Inc. 1992. Pakistan Demographic and Health Survey 1990-91. Islamabad, Pakistan: National Institute of Population Studies and Macro International Inc.
2. National Institute of Population Studies (NIPS) [Pakistan], and Macro International Inc. 2008. Pakistan Demographic and Health Survey 2006-07. Islamabad, Pakistan: National Institute of Population Studies and Macro International Inc.
